# Safety and tolerability of adjunctive rosiglitazone treatment for children with uncomplicated malaria

**DOI:** 10.1186/s12936-017-1858-0

**Published:** 2017-05-23

**Authors:** Rosauro Varo, Valerie M. Crowley, Antonio Sitoe, Lola Madrid, Lena Serghides, Rubao Bila, Helio Mucavele, Alfredo Mayor, Quique Bassat, Kevin C. Kain

**Affiliations:** 1 ISGlobal, Barcelona Institute for Global Health, Hospital Clínic, Universitat de Barcelona, Rosselló 132, 5th Floor, 08036 Barcelona, Spain; 2 Centro de Investigação em Saúde de Manhiça, Rua 12, Vila da Manhiça, 1929 Maputo, Mozambique; 3 S. A. Rotman Laboratories, Sandra Rotman Centre for Global Health, University Health Network-Toronto General Hospital, Toronto, Canada; 4 Toronto General Research Institute (TGRI), University Health Network, Toronto, Canada; 5 Women’s College Research Institute, Women’s College Hospital, Toronto, Canada; 6 Department of Immunology and Institute of Medical Sciences University of Toronto, Toronto, Canada; 7 ICREA, Pg. Lluís Companys 23, 08010 Barcelona, Spain; 8 Department of Medicine, University of Toronto, Toronto, ON Canada; 9 Tropical Diseases Unit, Division of Infectious Diseases, Department of Medicine, UHN-Toronto General Hospital, Toronto, ON Canada

## Abstract

**Background:**

Despite the widespread use and availability of rapidly acting anti-malarials, the fatality rate of severe malaria in sub-Saharan Africa remains high. Adjunctive therapies that target the host response to malaria infection may further decrease mortality over that of anti-malarial agents alone. Peroxisome proliferator-activated receptor-gamma agonists (e.g. rosiglitazone) have been shown to act on several pathways implicated in the pathogenesis of severe malaria and may improve clinical outcome as an adjunctive intervention.

**Methods:**

In this study, the safety and tolerability of adjunctive rosiglitazone in paediatric uncomplicated malaria infection was evaluated in Mozambique, as a prelude to its evaluation in a randomized controlled trial in paediatric severe malaria. The study was a prospective, randomized, double-blind, placebo-controlled, phase IIa trial of rosiglitazone (0.045 mg/kg/dose) twice daily for 4 days versus placebo as adjunctive treatment in addition to Mozambican standard of care (artemisinin combination therapy Coartem^®^) in children with uncomplicated malaria. The primary outcomes were tolerability and safety, including clinical, haematological, biochemical, and electrocardiographic evaluations.

**Results:**

Thirty children were enrolled: 20 were assigned to rosiglitazone and 10 to placebo. Rosiglitazone treatment did not induce hypoglycaemia nor significantly alter clinical, biochemical, haematological, or electrocardiographic parameters.

**Conclusions:**

Adjunctive rosiglitazone was safe and well-tolerated in children with uncomplicated malaria, permitting the extension of its evaluation as adjunctive therapy for severe malaria.

The trial is registered with Clinicaltrials.gov, NCT02694874

## Background

Malaria causes an estimated 212 million infections and 429,000 deaths annually [1]. Following the demonstration of the superiority of intravenous artesunate compared to quinine, artesunate has become the standard of care for severe malaria in both adults and children [2, 3]. However, in spite of its improved efficacy over quinine, case fatality rates for severe malaria remain high, ranging from 8.5 to 30% [2, 3]. In addition, substantial post-severe malaria morbidity persists with long-term neurocognitive impairments, such as deficits in attention, memory, speech, and language reported in up to one-third of children surviving severe malaria [4–14]. Both parasite and host determinants contribute to the pathobiology of severe malaria. The host immune response plays a central role in the onset, severity and outcome of malaria infections, and this has accelerated the search for immunomodulatory adjunctive therapies that could improve clinical outcome. To date, several putative adjunctive strategies have been tested in severe malaria, however with disappointing results [15, 16].

Peroxisome proliferator-activated receptor-gamma (PPARγ) is a member of the family of nuclear hormone receptors that function as ligand-activated transcription factors via their heterodimerization with another nuclear receptor, retinoic X receptor (RXR) [17–19]. PPARγ agonists are promising candidates for adjunctive malaria treatment as they have been reported to have anti-inflammatory, anti-oxidant, and neuroprotective properties [19–24]. The PPARγ agonist rosiglitazone is in the thiazolidinedione (TZD) class of drugs and is approved for the treatment of type II diabetes [25]. Rosiglitazone acts by increasing insulin sensitivity rather than increasing insulin levels, does not induce hypoglycaemia, and has an established safety profile in adults [25–29].

Rosiglitazone has been shown to enhance macrophage phagocytosis of *Plasmodium falciparum* parasitized erythrocytes, and to reduce parasite-induced pro-inflammatory cytokine secretion from monocytes and macrophages in vitro [30]. In a pre-clinical in vivo model of experimental cerebral malaria (ECM), rosiglitazone improved survival over artesunate alone, enhanced parasite clearance, reduced systemic inflammation and endothelial activation, prevented vascular leak, enhanced neuroprotective pathways, and protected mice from malaria-induced cognition and motor impairments [22, 31]. In light of these promising pre-clinical results, a randomized double-blind placebo controlled trial was conducted in young adults with uncomplicated malaria on the Thai–Cambodian border [32]. In this randomized trial, rosiglitazone was safe and well tolerated, and led to significantly improved parasite clearance times, lower levels of pro-inflammatory mediators, evidence of enhanced endothelial quiescence, and increased levels of the neuroprotective mediator brain-derived neurotropic factor (BDNF) [22, 32].

Together, these results support the hypothesis that adjunctive rosiglitazone may improve outcomes in patients with severe malaria. Since the majority of severe malaria and associated deaths occur in children under 5 years of age in sub-Saharan Africa, we conducted a phase IIa safety and tolerability trial in Mozambican children with uncomplicated malaria, as a prelude to undertaking a randomized trial in children with severe malaria.

## Methods

### Study design and participants

This was a prospective, parallel arm, unequally randomized, placebo-controlled, double-blind trial of rosiglitazone versus placebo, in 30 Mozambican children with uncomplicated malaria. All children received the Mozambican standard of care for uncomplicated malaria (Coartem^®^ Dispersible; artemether–lumefantrine 20 mg/120 mg, Novartis) with dosage determined by body weight, twice daily as recommended by National guidelines [33]. An unequal randomization list (2:1, in favour of rosiglitazone) was generated using blocks of 3, using the free online randomization software Sealed Envelope™ (https://www.sealedenvelope.com/). Randomization codes were placed inside individual sealed envelopes that were opened only by the nursing staff responsible for the administration of the drug. The remaining investigators were blind to the allocated intervention. All laboratory tests and statistical analyses were performed blinded to treatment group. Enrollment took place between February and March 2016.

### Ethical considerations

This study was reviewed and approved by the Mozambican National Bioethics Committee (CNBS) (Ref. 230/CNBS/15), the pharmaceutical department of the Mozambican Ministry of Health (Ref. 374/380/DF2016), the Clinical Research Ethics Committee of the Hospital Clínic, Barcelona, Spain (Ref. HCB/2015/0981), and the University Health Network Research Ethics Committee, Toronto, Canada (UHN REB Number 15-9013-AE). All research was conducted according to the principles expressed in the Declaration of Helsinki. The trial was registered with ClinicalTrials.gov on 9 December 2015, NCT02694874. All participants and their parents/legal guardians were given detailed oral and written information about the trial, and children were recruited only after a written informed consent was signed by their parents/legal guardians. Verbal assent was obtained from children over the age of 8.

### Study setting, inclusion and exclusion criteria

The trial was conducted by the *Centro de Investigação em Saúde de Manhiça* (CISM) at the Manhiça District Hospital (MDH), in southern Mozambique. A detailed description of CISM may be found elsewhere [34]. In Mozambique, malaria transmission is perennial, with a seasonal peak from November to April [35]. Parents/caregivers of children presenting to MDH were asked to participate in the trial and were screened for eligibility. Children, aged 1–12 years, were included in the study if they were positive for *P. falciparum* by microscopy, whereby a thick blood film confirmed malaria infection with parasitaemia >2500 parasites/μL. Children were excluded if they were known to have any known pre-existing illness (including neurological or neurodegenerative disorders, cardiac, renal or hepatic disease, diabetes, epilepsy, cerebral palsy), presented any reason for hospitalization, or if they had clinical or laboratory evidence of severe malaria (including severe anaemia, hypoglycaemia, acidosis, repeated seizures, prostration, impaired consciousness, respiratory distress, or age-adjusted tachypnea). Children receiving any therapy with potential anti-malarial activity (including cotrimoxazole), or treatment with a TZD were also excluded. Patients were approached after voluntarily presenting to MDH as part of routine care, and no financial incentives were provided.

### Intervention

Rosiglitazone (Avandia^®^, GlaxoSmithKline) and an identical looking placebo manufactured at the Hospital Clínic’s pharmacology department in Barcelona, Spain, were packaged and labelled to ensure blinding of study staff and hospital personnel. Children received either rosiglitazone (0.045 mg/kg/dose) or placebo twice daily for 4 days [36]. This dose was based on the maximal dose used by the manufacturer in the pediatric evaluation of rosiglitazone in children 10–17 [36]. The study medication was administered at the hospital, within the Clinical Trials Unit, by authorized members of the study team only. The study intervention (rosiglitazone or placebo) was started together with the first dose of artemether–lumefantrine. The interventions were administered orally. If any patient vomited or otherwise expelled the medication within 5 min of administration, the patient would be retreated. Rosiglitazone and placebo tablets were crushed and administrated as a powder mixed in water.

### Treatment follow-up and laboratory procedures

Following documentation of informed consent, participants had an initial targeted physical examination performed by the study physician. Anthropomorphic measures were calculated upon admission using the WHO AnthroPlus Software version 1.0.4 for children 0–19 years old [37]. A blood sample was taken at baseline and prior to the administration of the study intervention, for malaria diagnosis by microscopy, and haematological (haemoglobin, haematocrit, platelets, white cell full blood count) and biochemical (renal and liver function, glucose and lactate) evaluations. For a strict monitoring of glycaemia, finger-prick samples for glucose monitoring were obtained on admission, every 6 h for the first 48 h, and then every 24 h until discharge, and again at the day 7 and day 14 follow-up visits. Hypoglycaemia was defined as blood glucose <2.5 mmol/L (45 mg/dL) in an adequately-nourished child according to WHO definition [37]. Lactate was monitored on admission, every 12 h for the first 24 h, and then every 24 h until discharge, and again at the day 7 and day 14 follow-up visits. Biochemistry, including aspartate aminotransferase (AST), alanine aminotransferase (ALT), urea, creatinine, lactate dehydrogenase (LDH), and indirect and direct bilirubin, were assessed in venous blood every 24 h from admission until discharge and once again on day 7 follow-up. Venous blood extraction for haematology was performed every 24 h from admission until discharge and again on day 7 and 14 follow-up. Finally, venous blood extraction for biomarker analysis was performed on admission, 12, 24, 36, 48, 60, 72, and 84 h after admission, and again at the day 7 and 14 follow-up visits. Electrocardiographic monitoring was performed using a portable 12 lead electrocardiogram (ECG) machine (Cardioline ECG100+; AB Medica Group SA) at screening (before administration of study interventions), on day 1 (24 h after admission and after the second dose of study intervention), and on day 4 (after the last dose of study intervention). An additional ECG was conducted on day 7, only if abnormalities were recorded on day 4. The study clinicians reviewed all ECG tracings immediately after they were obtained, paying special attention to the QT segment length and potential prolongations from baseline. All children were kept at the health facility for the 4-day dosing period, despite being uncomplicated malaria cases. The mother/guardian was asked to return with the child for scheduled visits on day 7 and 14 post-treatment, or if any symptoms occurred. On each visit, a physical examination was performed by the study clinicians, vital signs were recorded, and body temperature measured.

### Outcomes

The primary outcome was safety and tolerability over the first 84 h of hospital admission as determined by clinical, biochemical, haematological, and electrocardiographic observations according to specific pre-defined local reference ranges. Adverse events (AEs) and serious adverse events (SAEs) were recorded and monitored throughout the study.

### Statistical analyses

Statistical analyses were performed with SPSS v.24 and Graph Pad Prism v.7. Differences between groups were assessed using the Fisher’s exact test for categorical demographic values, and by t test or Mann–Whitney U test (two-tailed) for clinical laboratory data based on the distribution of the data. Glucose measures at each time point were compared using a Mann–Whitney U test (two-tailed).

Mann–Whitney U tests were used to compare the median changes from baseline for glucose, AST, ALT, haemoglobin and haematocrit between the two treatment arms. Mean haematocrit and haemoglobin values were compared using ANOVA. A p value <0.05 was considered as statistically significant.

## Results

**Fig. 1:**
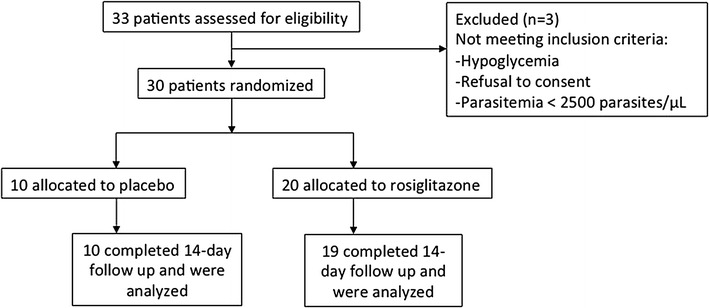
Study profile of 33 patients screened for eligibility for a randomized, placebo-controlled, phase IIa trial of adjunctive rosiglitazone for the treatment of uncomplicated malaria in children

The trial’s flow diagram is shown in Fig. 1. Baseline characteristics were similar between the two treatment groups (Table 1). Clinical monitoring of vital signs, including respiratory rate, heart rate, blood pressure, and oxygen saturation levels did not differ between groups. All study patients, irrespective of randomization group, had an eventless clinical course. No adverse drug reactions were observed, and no patient vomited in either group. No patient enrolled in this study had a SAE or died.

**Table 1:** Patient characteristics on admission

	Placebo, N = 10	Rosiglitazone, N = 20	p
Female sex (%)	7 (70%)	11 (55%)	0.70
Age (years)^a^	8 (4.6, 9.1)	6.9 (4.7, 9.8)	0.92
Weight (kg)	21.5 [15.0, 24.8]	20.2 [16.1, 2.7]	0.87
Height (cm)	124.0 [101.0, 132.5]	115.0 [106.0, 131.5]	0.75
BMI/age	−1.13 [−1.40, −0.37]	−0.15 [−3.2, 0.24]	0.11
Weight-for-age Z score (WAZ)	−1.1 [−1.4, −0.73]	−0.67 [−1.5, 0.81]	0.34
Temperature (^o^C)	38.3 [36.6, 39.1]	36.6 [36.1, 37.5]	0.07
Heart rate (bpm)	123.2 (15.58)	120.1 (14.6)	0.60
Glucose (mmol/L)	7.2 [5.3, 8.3]	5.9 [5.1, 7.3]	0.40
Lactate (mmol/L)	2.5 [2.1, 2.9]	1.9 [1.8, 3.2]	0.23
Haemoglobin (g/L)	10.1 (0.8)	10.1 (1.5)	0.99
Haematocrit (%)	30.1 (2.3)	30.4 (4.0)	0.83
Leukocytes (×10^9^/L)	8.3 (3.0)	7.4 (2.5)	0.41
Platelets (×10^9^/L)	96.3 (34.3)	131.8 (59.4)	0.09
AST (U/L)	53.8 (41.7)	36.4 (10.5)	0.08
ALT (U/L)	27.5 [5.3, 41.0]	33 [26, 36.8]	0.54
Urea (mg/dL)	12.5 [12.0, 17.3]	14.0 [13.0, 15.8]	0.67
Creatinine (µmol/L)	35.3 [30.7, 39.0]	31.2 [29.8, 42.8]	0.81
Parasite density (parasites/μL)	23,499 {4166, 55,491}	24,622 {3346, 91,869}	0.91

Median [IQR] for non-normally distributed variables

Number (%) for categorical variables

Parasite density is represented as geometric mean {range}

^a^Mean (SD) for normally distributed variables

**Fig. 2:**
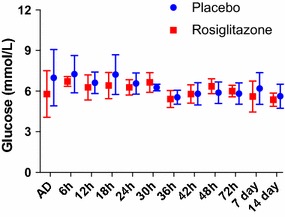
Median glucose levels of study participants on admission (AD) and at each measured time point.* Bars* represent interquartile range

Treatment with rosiglitazone did not induce hypoglycaemia, and all glucose were above the lower blood glucose threshold of 2.5 mmol/L (Fig. 2). Median changes in glucose levels between baseline and day 14 did not significantly differ between the placebo and rosiglitazone arms (1.2 vs. 0.3 mmol/L, p = 0.52). Only one isolated lactate value out of the normal range (0–5 mmol/L) was observed in a rosiglitazone-treated patient at 24 h post recruitment (5.5 mmol/L) that stabilized without any additional treatment.

Only two values of AST were out of range (0–100 U/L). These were observed at baseline in two children receiving placebo. They spontaneously normalized and were not associated with any clinical symptoms or signs. Median changes in AST levels between baseline and day 7 did not significantly differ between placebo and treatment arms (3.5 vs. 7 U, p = 0.78). Nor was there a significant difference in median changes in ALT between placebo and rosiglitazone treated participants (3.5 vs. 11 U, p = 0.15) between baseline and day 7. For the remaining biochemical parameters, including urea, creatinine, LDH, direct or indirect bilirubin, no significant differences or trending abnormalities were observed.

**Fig. 3:**
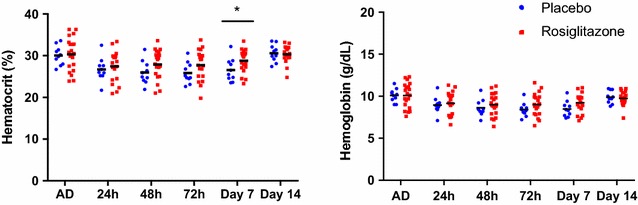
Mean hematocrit and hemoglobin levels according to study group on admission (AD) and at each measured time point

Haematological adverse events were uncommon. Three children had a haemoglobin decrease >2 g/dL from their baseline values, and further two had a haemoglobin value below the 7 g/dL threshold; none were associated with clinical manifestations. Median changes in haemoglobin levels between baseline and day 14 did not significantly differ between the placebo and rosiglitazone arms (0.4 vs. 0.7 g/L, p = 0.56). Median changes in haematocrit levels between baseline and day 14 did not significantly differ between the placebo and rosiglitazone arms (0.4 vs. 0.7 g/L, p = 0.56). There was no significant difference between the treatment groups on haematocrit or haemoglobin levels (p = 0.13 and p = 0.12, respectively, Fig. 3). No differences in haematological parameters, including leukocytes, platelets or other components of the complete blood cell count, were observed between groups.

Table 2 summarizes all ECG abnormalities found during the study follow up. None of these events were associated with clinical findings or with the study intervention. No additional medication was administered to these patients. No patient had a QTc of more than 500 ms at any of the measured time points.

**Table 2:** ECG abnormalities

Patient	Treatment	QTc baseline (ms)	QTc maximum (ms)	ECG findings
ROSI-002	Rosiglitazone	352	407	Increase of QtcF >50 ms on day 4, NCS
ROSI-007	Rosiglitazone	396	427	Increase of QtcF >50 ms on day 2, NCS. Finished on day 4
ROSI-008	Rosiglitazone	356	424	Increase of QtcF >50 ms on day 2, NCS. Continue on day 7, NCS
ROSI-009	Placebo	342	425	Increase of QtcF >50 ms on day 4 with associated bradycardia, NCS Increase of QtcF >50 ms on day 7, NCS and without bradycardia
ROSI-010	Placebo	336	409	Increase of QtcF >50 ms on day 4, NCS. Finished on day 7
ROSI-012	Rosiglitazone	323	404	Increase of QtcF >50 ms on day 2, NCS. Finished on day 4
ROSI-014	Placebo	364	425	Left bundle branch block from screening, NCS Increase of QtcF >50 ms on day 4, NCS. Finished on day 14 Bradycardia on day 4, NCS
ROSI-021	Placebo	361	414	Increase of QtcF >50 ms on day 4, NCS. Finished on day 7
ROSI-022	Placebo	349	402	Increase of QtcF >50 ms on day 4, NCS. Finished on day 7

These abnormalities were not clinically significant (NCS). QT corrected for heart rate using Fridericia’s method

**Fig. 4:**
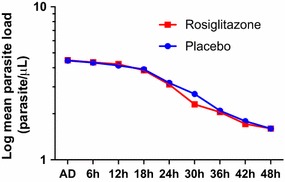
Log mean parasite load in Mozambican children with uncomplicated malaria according to study group

Mean parasite densities at baseline and throughout the study were similar between groups, (Fig. 4). Median time to parasite clearance was 33 h in both the rosiglitazone and placebo groups (p = 0.88).

## Discussion

In this study, the safety and tolerability of rosiglitazone as an adjunctive therapy for the treatment of uncomplicated malaria was investigated, as a first step before evaluating its efficacy in the treatment of severe paediatric malaria and prevention of malaria-associated neurocognitive complications. Although rosiglitazone has a well-established safety profile, and millions of doses have been administered to adults with type II diabetes mellitus [38], this was the first time that the drug was used (off-label) as an adjunct to standard malaria treatment in African children. For this reason, and in concordance with recommendations issued by the CNBS, 30 children with uncomplicated malaria were carefully followed, and assessed for a variety of haematological, biochemical, clinical, and electrocardiographic safety variables, to ensure that the drug was not associated with adverse events. Although rosiglitazone was found to be safe and well-tolerated in children with uncomplicated malaria it cannot be assumed that a similar safety profile would be observed in children with severe malaria. Any future studies investigating the use of rosiglitazone for this purpose will be required to include additional safety evaluations.

Hypoglycaemia is a common and life-threatening complication of malaria [39], and drugs with negative effects on glycaemia levels would be problematic as adjuvant malaria therapies. Thus, particular attention was placed in assessing whether rosiglitazone would be associated with any glycaemia abnormalities in this population. Rosiglitazone acts by increasing insulin sensitivity rather than increasing insulin levels, and therefore, it is unlikely to induce hypoglycaemia. Although our sample size was small, it was reassuring that none of the malaria infected participants displayed glycaemia levels below 2.5 mmol/L, and glycaemia levels were similar between the treatment and placebo groups.

A second important aspect of this trial was to assess potential adverse cardiac events. Initial case–control studies of long-term use in elderly high-risk diabetic patients reported a potential increase in acute cardiac events [28]; however these studies had methodological problems and recent reports from the USA Food and Drug Administration (FDA) have concluded that rosiglitazone is safe and was not associated with excess cardiovascular risk [28]. Importantly, as of June 2013, an FDA expert panel relaxed restrictions on this drug [29]. In agreement with these findings, no cardiac adverse events or increased risk over placebo were observed in a previous randomized trial in adults with uncomplicated malaria [32]. Similarly, and as anticipated for a paediatric non-diabetic patient population treated with a short course (4 days) of rosiglitazone, no cardiovascular adverse events were observed in this study. Additionally, some oral artemisinin-based combinations have been reported to induce prolongation of the electrocardiogram’s QT interval, while malaria infection itself can increase the sympathetic tone of the heart, which manifests as a shortening of the QT interval in ECG traces [40]. Repeated ECG measurements detected only a few electrocardiographic abnormalities, none of which were deemed related to the investigational drug. The incidence and clinical significance of these electrocardiographic abnormalities were equally distributed between the placebo and rosiglitazone groups.

Rosiglitazone has been reported to decrease mean haemoglobin and haematocrit in a dose-related fashion in adults, particularly when it is taken on a daily basis [41]. This potential effect needed to be evaluated in the context of malaria, a disease often associated with decreases in haemoglobin levels. No major declines in haemoglobin or haematocrit were observed in either study arm, and no significant interaction between the study arm and sampling time were observed for either haemoglobin or haematocrit.

Rosiglitazone, malaria, and many anti-malarial drugs can be associated with increases in hepatic transaminases [41–44]. In this trial, transaminase levels were monitored in addition to direct and indirect bilirubin, and no patient receiving rosiglitazone had levels outside of the normal range.

This study was not powered to evaluate efficacy endpoints of rosiglitazone. As such, the outcomes of the uncomplicated malaria participants recruited to evaluate the safety and tolerability of the drug, may not be sufficiently informative of the potential that the drug has to impact the course of disease. Although increase parasite clearance times were previously observed in adult patients randomized to rosiglitazone in a previous randomized control trial these patients received atovaquone/proguanil [32]. Even with a study powered to study efficacy we may not see improved parasite clearance times due to the fast clearance of early rings by artemisinins. There is limited pharmacokinetic–pharmacodynamic data of rosiglitazone in children under the age of 10. These data would have added more reassurance regarding the safety of rosiglitazone in children and is a limitation of the study. Further evaluation of rosiglitazone, when used as an adjunctive therapy in the context of severe malaria, will be required to explore its impact on pathways implicated in the pathogenesis and outcome of severe malaria.

## Conclusion

This is the first report of rosiglitazone use in African children with an acute uncomplicated malaria infection. The safety and tolerability results, including no vomiting, no idiosyncratic drug reactions, and no serious adverse events support its continued evaluation as an adjuvant therapy in the treatment of severe paediatric malaria.

## Abbreviations

ACT: artemisinin-based combination therapy
AE: adverse events
ALT: alanine aminotransferase
Ang-2: angiopoeitin-2
AST: aspartate aminotransferase
BDNF: brain-derived neurotropic factor
CISM: Centro de Investigação em Saúde de Manhiça
CNBS: Mozambican National Bioethics Committee
ECM: experimental cerebral malaria
LDH: lactate dehydrogenase
MDH: Manhiça’s District Hospital
ms: milliseconds
PPARγ: peroxisome proliferator-activated receptor-gamma
RXR: retinoic X receptor
SAE: serious adverse event
TZD: thiazolidinedione
